# The Use of Cold and Heat in Fevers

**Published:** 1878-04-20

**Authors:** Frank Alport

**Affiliations:** Sycamore, Ill.


### THE USE OF COLD AND HEAT IN FEVERS.

By Frank Alport, M.D., Sycamore, Ill.

In the treatment of the febrile state there are two methods of procedure, one of which is endorsed by a large portion of the profession both in this country and Europe, the other is struggling for a position, and already numbers among its advocates some of the best names known to medicine. They are apparently so diametrically opposite in their action as to excite the inquiry why they can both be used in dealing with the same affections. I have reference to the external application of cold and of heat. So pertinent indeed is the query that it is well worth while to stop and consider if the results of their employment may not be similar, although the means used are so different.

Internal heat is one of the most important of all the factors concerned in fevers of every description, and is invariably present. The extent to which it is found in each individual case militates largely for or against the recovery of the patient. It is therefore of great moment that some means be found by which we may nearly or quite free the system of this exalted internal temperature; bearing in mind always that *heat* is not the disease, but the result of the retention within the body of the products of excretion.

Probably more than to medicine the profession looks to either one of these remedies (cold or heat) to bring about this end. Let us then briefly analyze the action of each, after which we may, by a comparison of their merits and demerits, arrive at a conclusion as to which we may look upon with the greatest reliance for the accomplishment of the object in view. First, then, as to cold. The first effect produced is a shock, which for the time being paralyzes the nerves controlling the vascular system, throws the blood to the viscera, closes the pores of the skin and exalts the internal temperature. In a short time a reaction generally sets in. The blood flows to the surface and relieves visceral congestion, the pores of the skin are opened, a gentle perspiration suffuses the body, and the temperature is reduced a certain number of degrees. Very soon a counteraction commences, the blood recedes from the surface and the original, or even more than the original, degree of internal heat is present.

Upon the application of heat the action on the system is about as follows: In the first place, the blood gradually leaves the viscera and seeks the surface. The pores are opened and a perspiration breaks forth, which soon becomes profuse, and, as the system is emptied of the accumulated poisons, the temperature is reduced a certain number of degrees. After the heat is reduced and the perspiration limited, the temperature of the body again runs up to or within one or two degrees of the original, sometimes equalling it; but under proper management, in a fairly commenced case of fever, where the disease terminated favorably, I have never seen it exceed it. These may be said to be the respective actions of the two remedies.

It will be observed, that whereas with cold, shock and exaltation of temperature, are the effects produced; with heat there is no shock, no exaltation of temperature, and the good results at once become manifest. I have said in reference to cold, "a reaction *generally* sets in." I have heard and read of cases where patients never recovered; death occurring from the shock. The reaction then, does not always occur, in which case death is the result. It is reaction therefore, which does the good, indeed which saves the patient's life from the first effects of the remedy, viz: closure of the cutaneous pores, shock, and increased visceral congestion. If I may use a non-medical illustration, the application of cold, is like the experiment of Mr. Squeers, who almost knocks poor Smike off the trunk with one hand, and saves him from falling with the other. It is not then to the immediate effect of cold to which the benefit may be ascribed, but to the powerful recoil of the system, from the shock produced by the application of cold.

To bear me out in these assertions, I will quote the words of the editor of the *British Medical Journal*, while speaking of the external application of cold in fevers: "The only contra-indications to this treatment, are, first of all, hemorrhage from the bowels in typhoid fever, even if it be present in the slightest degree, perforation also precludes its continuance." These sentiments are from the pen of an advocate of this method of procedure, and they would seem to show, that notwithstanding a reaction may take place, it is erroneous practice to plunge the patient into a cold affusion, thus still further congesting the already highly inflamed viscera. In the treatment of the febriculæ, we must bear one idea constantly in mind, viz: at present we have no specifics for this state, and we must treat the symptoms. The patient should be placed in the best possible general condition to recover. If any organ or organs are found to be acting improperly, the error should be corrected. Perhaps more important than all, we must see to it, that the secretory and eliminative organs are acting with a normal, and even more than a normal degree of activity. Therefore, when we see, as we do, almost if not quite invariably, in the febrile state, that while more urea, carbonic acid, and other eliminative materials are produced, yet the quantity of these substances cast off, is *less* than in health, we must aim, by every possible means, to correct the error.

No one can doubt the usefulness of the skin as an excretory organ, in this predicament, and to it we should direct our attention first. I say "first" because the kidneys, bladder, etc., are in such a congested and diseased condition, that they are largely incapacitated for work, and only by a vicarious elimination can they be relieved of this state, and rendered equal to the task assigned them.

Dr. Davis says that the only two cases of complete suppression of urine which have ever occurred in his own practice, following scarlet fever, were benefited only by those remedies which promoted the elimination of the retained elements through the skin, kidneys, and bowels. In these severe cases, specifics were set aside, and only such means adopted as were directed to placing the system in the nearest to a normal condition possible, viz: by looking to the excretions. If such treatment is correct in the severer cases, why is it not just as reliable in milder ones? And yet how often do practitioners tamper in the beginning of fevers with so-called "specifics," etc., who are compelled, when the disease becomes of an alarming type, to resort to remedies controlling elimination.

Considering the foregoing facts, we are led to conclude, that profuse and steady perspiration is a most desirable and important end to be attained.

It would appear that fever is simply a state of innervation produced by the septic influences of a (possible) "fever-poison," and kept alive by the non-elimination of natural and abnormal elements of excretion that should be cast off. The delirium and coma of scarlet fever are, then, simply symptoms produced by this innervated state, and the retention and circulation in the tissues of the brain of the manufactured, but not excreted poisons.

A comparison of the reduction of temperature, produced respectively by the application of external cold or heat, shows that the former is the most decided in its action. Dr. Beal cites cases from the practice of Dr. Wilson Fox, where the temperature was reduced by a single immersion, eleven degrees. The reduction by heat has never, so far as I can learn, equalled this by several degrees. This might at first be deemed an argument in favor of cold. On the contrary, it is one against it, for there can be no doubt that such rapid and eccentric changes are generally of positive injury. We must bear in mind that, after this excessive reduction of temperature, a counteraction takes place, and the thermometer will in a very short time register a rise of as many, and generally more than as many, degrees as were previously lost. Such decided and sudden changes cannot be of benefit. What we want is a gradual but sure declination of temperature, such as we obtain from the proper application of external heat. The temperature in this mode of treatment, under proper circumstances, and if the "sweat" is given often enough, rarely returns to its former height, but will register a sure loss, although it is not so rapid as in the treatment by cold. In this particular I would say that Dr. T. Clifford Albutt, A. M., an advocate of the "cold bath treatment," states that a too rapid diminution of temperature is positively injurious in fevers. With this idea in mind, he endeavors, while still clinging to the "cold bath," to somewhat modify the application of it, in order that so rapid a diminution may not be produced. Another reason why the "cold bath" should not be used, is that the patient requires too much careful watching, such as it is almost impossible for a physician in full practice to give. Again, Dr. Albutt says: "I would urge the continual presence of a medical man. The whole treatment must be managed with the utmost precision, and all tendencies to shiver or syncope, should be watched by a skillful observer, or irreparable damage may be done in five minutes." Now it is evident that only in a hospital can a physician be constantly with his patient (and even there it would be difficult), to act *immediately* upon the approach of certain symtoms, and a physician would hardly feel justified in placing such a powerful and easily fatal remedy in the hands of the laity. Is it then proper to continue the use of this remedy, so potent for evil, while we have, in heat, an agent capable of doing more good, with no practical risks, provided the patient receives reasonable care from a competent nurse, in the absence of the physician?

A comparison of the merits and demerits of the external application of heat or cold, in the treatment of fevers, may assist in reaching a conclusion as to which is to be preferred, and for this purpose I submit the following table:

| *Heat.* | *Cold.* |
| --- | --- |
| No shock is experienced upon applying the remedy. | A shock is experienced. |
| Perspiration soon follows the application of heat. | Perspiration does not take place for some time after cold has been applied, *and it is dependent upon a reaction,* which may or may not take place. In the latter event, a fatal result is apt to occur. |
| A gradual declination of temperature is observed. | A sudden and great declination of temperature is observed. |
| The temperature is not apt to rise above that registered at the commencement of the application, after the same. | The reverse is the rule. |
| Profuse perspiration is produced, and it does not require to keep the temperature down, as many applications per diem as with the application of cold. | Only a gentle perspiration is produced. |
| Only a reasonable amount of care and watching is required. | Directly the reverse is true. |

—*Chicago Medical Examiner.*

